# The New Pharmacopœia and the Proprietaries

**Published:** 1906-03

**Authors:** Samuel A. Visanska

**Affiliations:** Atlanta, Ga.


### THE NEW PHARMACOPOEIA AND THE PROPRIETARIES.*

By SAMUEL A. VISANSKA, Ph.G., M.D.,

Atlanta, Ga.

Perhaps many of you will think that I have returned to my first love, pharmacy, when I attempt to bring out for discussion a few of the changes in the new United States Pharmacopoeia; but the changes have been so sweeping as to the strength, nomenclature, drugs admitted and dropped, etc., that I thought the sooner we become familiar with them, the better it will be for both physician and druggist. I would not be so cruel as to attempt to mention all the changes, and every drug or preparation which this change affects, but will refer you to this most excellent book, the eighth decennial revision of the United States Pharmacopoeia.

The Pharmacopoeia is revised every ten years by a committee on revision, which is an integral part of a chartered organization, the United States Pharmacopoeial convention.

*Scope of the Pharmacopoeia.*—The committee of revision was authorized to admit into the Pharmacopoeia any product of a nature of known origin, also any synthetized product of definite composition which is in common use by the medical profession, the identity, purity or strength of which can be determined; no compound or mixture shall be introduced, if the composition or mode of manufacture be kept secret, or if it be controlled by unlimited proprietary or patent rights.

*Doses.*—For the first time, the committee saw fit to place after

*Read before the Fulton County Medical Society.

each drug, chemical or preparation intended for internal use, the average approximate dose (but neither a minimum or maximum dose) for adults, and when deemed advisable, also for children. It was distinctly understood that neither the convention nor the committee on revision created by it intended to have the doses as obligatory on the physician, or as forbidding him to exceed them whenever, in his judgment, it seemed advisable.

*Nomenclature.*—It was recommended that changes in the titles of articles at present official be made only for the purpose of insuring greater accuracy, or safety in dispensing. One of the most important changes is that of the Latin for fluid extracts. This in previous revisions of the Pharmacopaeia was, for example, *extractum Rhei fluidum*, which compelled the intermingling in the extracts of ex tracts and fluid extracts, thus *extractum Rhei* was placed immediately before *extractum Rhei fluidum.* In this revision they are found under the letter F, *Fluidextractum* being now one word. The metric system was adopted, and the approximate equivalent of ordinary weights or measures inserted in parenthesis. The effort which has been made for years to establish an International Pharmacopoeia has been abandoned, but an important conference was held in the city of Brussels, September, 1902, which was composed of delegates from nearly every civilized country. The purpose of this body was to endeavor to formulate standards for potent remedies which would be adopted by the various pharmacopoeias of the world, and thus there would be secured the principal object of an International Pharmacopoeia. The recommendations of this conference have been adopted by the Committee on Revision, except in a few instances. This, therefore, has made necessary a number of changes in the strength of important official preparations. The adoption of the uniform strength of 10 per cent. for the tinctures of potent drugs has compelled the alteration in strength of many of the tinctures of the U. S. P. of 1890. Tr. Aconite is now 10 per cent., instead of 35 per cent.; Tr. Veratrum Viride 10 per cent., instead of 40 per cent., etc. The standard of 1 per cent. for the liquid arsenical preparations, and the long-established U. S. P. standard for fluid extracts (1 c.c. represents 1 gr. of drug.) were both adopted by the international conference. The strength of Syr. Ferrius Iodide has been reduced from 10 per cent. to 5 per cent.; Tr. Digitalis from 15 per cent. to 10 per cent.; Tr. Strophanthus increased from 5 per cent. to 10 per cent.; Basham's Mixture increased to double strength. Of the list of articles previously official, 151 have been dismissed, while 117 new ones have been introduced.

The extensive use of serum products since the last revision made it necessary for the committee to thoroughly consider the necessity for the introduction of the most important of these; namely, Serum Antidiphthericum. Two new animal products, Glandulae Thyroidae Siccae and Glandulae Suprarenalis Siccae have been introduced. A comparison of the number of articles, test solutions and assays shows that there are 1,297 in the present Pharmacopoeia against 1,257 in the previous one. Elixir of Phosphate of Iron, Quinine and Strychnine has been increased. Again, 3, Scopalamine Hydrobromate, dose $\frac{1}{128}$ grain, an alkaloid given with the Morphine as an aid to anesthesia. Phosphate and Sulphate of Codeine, Antipyrinum, Emulsion of Cod Liver Oil with Hypophosphites, Liquor Antisepticus, Carbonate of Guiacol, Pul. Acetanilid Co:, Syr. Hypophos. Co. Cataplas of Kaolin, have been admitted. This leads us to the practical part of my paper. The new Pharmacopoeia, for several reasons, did not go into effect until September, 1905. Now the point is just this: unless we have a distinct understanding with the druggists of our city, you will not know what strength you are getting when ordering a preparation. It would make quite a difference if you ordered Tr. Veratrum Vir. with the expectation of getting the 40 per cent. Tr., and you got the 10 per cent. Tr. You will be compelled in the future to specify U. S. P. 1890 or 1900. To obviate this trouble I would suggest a joint meeting of the Atlanta Druggists' Association and the Fulton County Medical Society; in fact, it would be a good idea to invite every druggist and physician who is interested; we could then discuss the subject I have just mentioned, and no doubt a specific date could be arranged when these changes will take place in Atlanta. The way the matter stands now, it is almost dangerous to prescribe. Your results depend on the strength and purity of the different preparations; the reliable druggist is your safeguard. I would like to see the druggists and physicians brought in closer touch with each other; one depends so much on the other, and there is no better time to bring this about than now.

DISCUSSION OF DR. VISANSKA'S PAPER.

*Dr. L. B. Clarke:* I regret Dr. Todd's absence from the meeting, inasmuch as he is far more able to discuss this paper than I am, and again, I regret that Dr. Visanska has left me very little to say, in not only having covered the ground completely, but that I agree with him in the main in what he has stated.

There are differences of opinion as to the advisability of the radical changes that have been made by the revisers of the new Pharmacopoeia, and we must concede to the objectors a certain amount of tenable ground, when we consider the reduction of the strength of the potent tinctures; for instance, Tinct. Aconite from 35 per cent. to 10 per cent. and Tinct. Veratrum from 40 per cent. to 10 cent. These are marked changes in two preparations in constant daily use and, of course, are likely to lead to great confusion. Until thorough accord exists between the physician and the druggist the former may be using a half or one drop dose of Tinct. Aconite, with absolutely no results, or vice versa; it is possible that he shall use a dangerous dose of either drug, under the supposition that he is only using the ten per cent. tincture.

Another objection that I personally see is the removal of a certain safeguard about these poisons hitherto existent in their minute dosage, and the necessary increase of dose three or four fold, which may—probably will—have a tendency to create less respect for their toxic properties.

The remedy for these evils has been suggested by Dr. Visanska in his idea that it would be well for the prescriber to designate U. S. P. 1890, or U. S. P. 1900, as the case may be.

The bulk of the changes in strength are not so extreme, fortunately, as the two above-mentioned drugs; the general change being 15 per cent. tinctures reduced to 10 per cent. and 5 per cent. increased to 10.

The eventual outcome is no doubt good, but years must necessarily elapse before the changes are all perfectly taught, understood and observed in thorough accord.

The object of the Pharmacopoeia is to standardize the purity and strength of preparations used medically; those found obsolete are thrown out at the revisions, and other preparations found gaining more and more popular approval among the physicians are added to it, with a definite standard formula for their manufacture. This is probably the reason for the addition of Emplastrum Kaoli Compos., which the pharmacist at once recognizes is identical with Antiphlogistine.

Now many of us use Antiphlogistine and regard it of great value, so the revisers say, "Well, if you all use it, we will put in a formula and let the druggist have his guide to its manufacture." Upon the other hand, it is possible that this addition will be used by the manufacturers of Antiphlogistine as an advertising card of the first magnitude in a statement that the Pharmacopoeia attests the value of their preparation by adopting it.

Finally, I agree with the proposition of Dr. Visanska that a joint meeting of the Fulton County Medical Society and the Atlanta Druggists' Association would prove of great value in eliminating many of these vexatious questions.

*Read before the Fulton County Medical Society.

